# Picornavirus Identified in Alzheimer’s Disease Brains: A Pathogenic Path?

**DOI:** 10.3233/ADR-200174

**Published:** 2020-05-20

**Authors:** Bo Niklasson, Lars Lindquist, William Klitz, Netherlands Brain Bank, Elisabet Englund

**Affiliations:** a Jordbro Primary Health Care Center, Stockholm, Sweden; b Department of Medicine Huddinge, Division of Infectious Diseases, Karolinska Institutet, Stockholm, Sweden; c Department of Integrative Biology, University of California, Berkeley, CA, USA; d Netherlands Brain Bank, Netherlands Institute for Neuroscience, Amsterdam, The Netherlands; e Department of Clinical Sciences, Division of Pathology, University of Lund, Lund, Sweden

**Keywords:** Alzheimer’s disease, immunohistochemistry, Ljungan virus, neurodegenerative disease, picornavirus

## Abstract

We investigated formalin-fixed postmortem brain tissue from the hippocampus region of 18 AD cases and 11 age-matched controls using a polyclonal antibody against Ljungan virus (LV) capsid protein 1. Evidence of a LV antigen was found in all AD cases but in none of the control specimens (*p* < 0.0001). The antibodies reacted with neurons and astrocytes and also showed distinct positive reaction in the amyloid/neuritic plaques. The possible role of an incompletely characterized picornavirus as the etiologic agent in AD open up the possibility of treatment with antiviral therapy directed against picornaviruses. The positive result of such treatment in a small number of patients is presented separately back to back to this report.

## INTRODUCTION

Alzheimer’s disease (AD) is globally known to be a chronic neurodegenerative disorder characterized by progressively deteriorating cognitive function where symptoms gradually worsen and the ability to communicate and perform daily activities ultimately become severely impaired. Biochemical and neuropathological studies of brains from individuals with AD provide clear evidence for an activation of inflammatory pathways [1].

Many viruses have developed the strategy of prolonged coexistence and establishment of persistent infections in their human host [2, 3]. Several microbial agents such as herpes simplex virus type 1, Helicobacter pylori, Chlamydia pneumoniae, and Lyme-Borreliosis, have been associated with neurodegeneration in either humans or in experimental animal models and are all implicated as possible causative agents for AD in humans [4–7]. Genome investigations have also identified a genetic signature that might affect individual brain susceptibility to infection by the herpes virus family during aging, leading to neuronal loss, inflammation, and amyloid deposition [5]. However, the compiled evidence linking AD with the infectious agents, although strong, is still incompletely understood.

We have earlier reported the finding of a novel picornavirus named Ljungan virus (LV), present in wild rodents and the association of this virus with diabetes, both in its wild rodent reservoir and in a type 1 diabetes laboratory rat model [8, 9]. Virus isolation, serology, and immunohistochemistry have been used to identify infected individuals and detect presence of virus in infected tissue [10, 11].

LV has also been associated with neurological disease in both humans and in laboratory animals. The virus causes intrauterine fetal death and neurological malformation in laboratory mice and has also been associated with intrauterine death and hydrocephalus in humans [12–14].

Recently we have extended our studies on LV to include neurodegenerative diseases using immunohistochemistry (IHC). We here report the finding of a LV related antigen in postmortem brain of patients with AD.

## MATERIALS AND METHODS

### AD patients analyzed for presence of viral antigen in brain tissue using immunohistochemistry

Brain samples from 3 AD cases and 2 controls came from the brain bank at the Department of Oncology and Pathology, University of Lund, while 15 AD cases and 9 controls came from the Netherlands Brain Bank. Male-female ratios for the AD patients were 1 : 1.1 with mean age of 74 years. The AD specimens came from deceased individuals clinically diagnosed based on clinical criteria [15]. These criteria require the presence of cognitive impairment and a suspected dementia syndrome confirmed by neuropsychological testing. All AD cases included in this study were histopathologically confirmed by IHC staining positive for hyperphosphorylated tau, alpha-synuclein protein, and histochemical positive staining for amyloid with alcalic congo [16, 17]. The neuropathological changes in the AD group were graded as Braak 4 (*n* = 1), Braak 5 (*n* = 5), and the remaining Braak 6 (*n* = 12) [18].

Control specimens came from patients with no history of cognitive impairment or suspected dementia syndrome and no postmortem histopathological signs of neurodegenerative disease. Male:female ratio in the control group was 1 : 1.3 mean age 77 years.

Formalin-fixed paraffin-embedded brain tissue from the hippocampus region was sectioned at 5 micrometers and analyzed for presence of LV antigen using IHC previously described, with minor modifications [19]. Presence of LV specific antigen was visualized using a polyclonal rabbit antiserum produced against bacterially expressed recombinant viral capsid proteins (VP 1) of the LV strain, 87-012G [20]. The IHC assay used Novocastra (RE7119) Epitope Retrieval Solution pH 9.0. As control, we used serum from a rabbit immunized using the same protocol but with the carrier protein only. Tissues from LV-infected and non-infected animals were included as additional controls. The specificities of the rabbit antibodies were verified by analyzing control specimens generated by mixing infected tissue culture cells with non-infected cells followed by formalin fixation and paraffin embedding. The specificity of the reaction was also confirmed by blocking the signal with LV antigen in parallel with control antigen.

## RESULTS

### LV antigen in brain tissue using immunohistochemistry

LV viral antigen was detected in the hippocampal sections of all 18 cases with AD but in none of the 11 age-matched controls. A control serum consisting of normal rabbit was negative in all 18 AD cases and 11 controls. The association of viral antigen presence in AD patients versus controls were found to be significant (*p* < 0.0001 Fischer’s exact two sided test).

**Fig.1:**
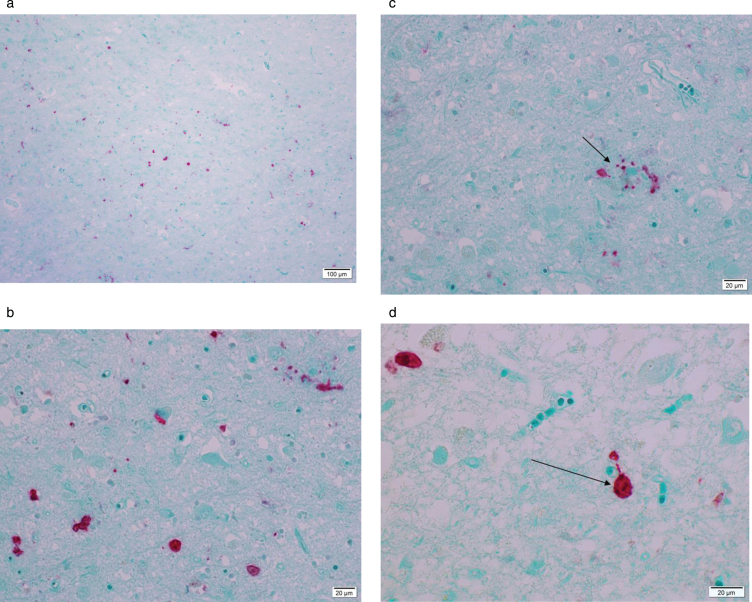
Microphotograph of formalin-fixed tissue from a 79-year-old patient diagnosed with Alzheimer’s disease staining positive using rabbit anti-Ljungan virus VP1 antibodies. Red stain visualizes presence of viral antigen. Panel A is an overview and Panel B is a magnification of the same region of the hippocampus. Red staining marks presence of viral antigen in neurons, astrocytes, and glial cells. In Panel C, an amyloid/neuritic plaque (marked with an arrow) in shown staining positive in the glial compartment and in dystrophic neurites. Panel D shows positive staining in neurons (marked with an arrow).

Figure 1 A–D are microphotographs of hippocampus tissue from one 79-year-old AD patient representative of the findings in all AD patients in this study.

**Fig.2:**
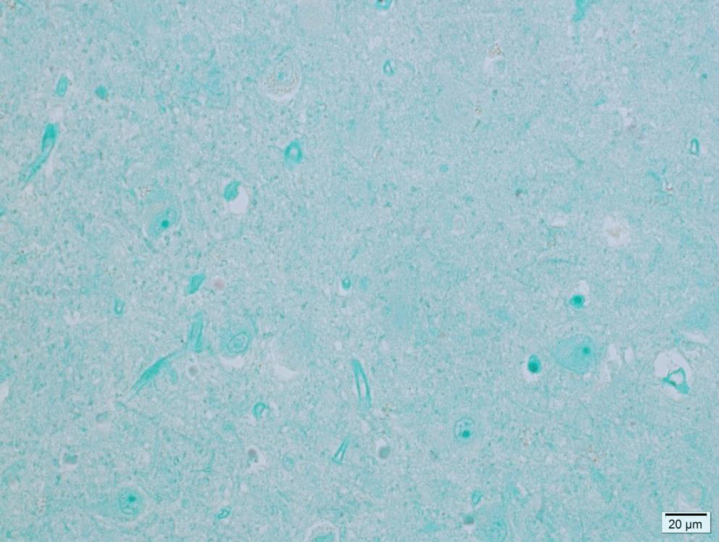
Microphotograph of formalin-fixed hippocampus tissue from a 78-year-old control patient showing no viral antigen staining.

The binding of rabbit anti LV VP1 antigen is seen as red stain showing distinct reactions in most cell types—neurons, astrocytes, and microglial cells. An amyloid plaque with positive viral antigen staining in both the glial compartment and in dystrophic neurites is illustrated in Figure 1 C. Figure 1D is a magnification of a viral antigen positive neuron from the same patient. No antigen positive cells were found in any if the 11 control patients. Figure 2 is a photomicrograph of the hippocampus from a 78-year-old individual from the control group exemplifying the absence of LV antigen staining among the controls.

## DISCUSSION

AD is associated with neuronal loss and progressive synaptic dysfunction, preceded by the deposition of amyloid-*β* (A*β*) peptide, a cleavage product of the amyloid-*β* protein precursor, and abnormal forms of tau protein, markers that have been used as diagnostic criteria for AD [21]. Although these markers constitute the hallmarks of the disease, their role in the pathogenesis of AD is not yet established. In fact, they are seen, with slightly varying forms of presentation, in a variety of other CNS conditions, including chronic infections. Neurofibrillary tangles and neuropil threads have been observed in cases of measles, virus-induced subacute sclerosing pan-encephalitis [22], and in tertiary syphilis [23]. Amyloid plaques are often present in cases of prion diseases such as Creutzfeldt-Jakob disease [24]. In addition, amyloid deposition and aggregation in tissues is a frequent occurrence in several acute and chronic systemic inflammatory conditions such as chronic infections caused by tuberculosis and leprosy [25, 26]. Furthermore, A*β* has been shown to have inherent antimicrobial properties, which further supports the possibility that A*β* production and deposition in AD might be induced by infectious pathogens [27]. Stress has been shown to account for tau protein brain dysfunction, as the second major neuropathological component of AD [28]. These recent observations recognizing the importance of antimicrobial function of A*β* and stress-induced abnormal phosphorylation of tau protein for the pathology of AD form a logical base for our study hypothesis.

It has been proposed that lifelong viral persistence in otherwise immunocompetent individuals may accelerate the aging of the immune system and may lead to chronic subclinical inflammation [29]. This may be one reason for an age-related decline in immune functions that contribute to the increased susceptibility of elderly persons to infectious diseases and vaccine failure [30]. This may also be the reason why a persistent infection previously under control without signs and symptoms slowly transform to an infection with severe tissue damage and devastating outcome for the infected organ and for the individual. Our findings give additional support for the hypothetic viral involvement in AD either by direct interference with neuronal function or by increasing other stress factors contributing to the pathogenesis of AD.

In man, the virus family Picornaviridae causes the widest range of diseases of all virus families. Infection with various picornaviruses may be asymptomatic or may cause clinical syndromes such as the common cold, febrile rash illnesses, conjunctivitis, hepatitis, myositis, and myocarditis [31]. Many picornaviruses have also been shown to have the ability to infect the CNS and cause various neurological symptoms, such as meningo-encephalitis and myelitis. Among these viruses, poliovirus is best known, while other non-polio picornaviruses account for approximately one half of aseptic meningitis cases in children [32]. Mice infected with Theiler’s Murine Encephalomyelitis Virus (TMEV), a close relative to LV, cause inflammation, demyelination, and neural damage resulting in disrupted spatial memory when tested using the Morris water maze test. Importantly, the degree of memory impairment correlated with hippocampal injury in this animal model [33].

LV, a member of the Parechovirus genus in the family Picornaviridae, was isolated from one of its wild rodent reservoirs, the bank vole (*Myodes glareolus*), near the Ljungan River in central Sweden [10]. LV infected bank voles in captivity develop several different pathological signs and symptoms including myocarditis, diabetes, encephalitis, and stereotypic behavior. Studies on laboratory mice showed that more than half of the females infected with LV during pregnancy and exposed to stress gave birth to pups that died during the perinatal period. Malformation of the CNS including hydrocephalus and anencephaly was seen in some of the offspring. Suckling mice infected during the first two days developed severe encephalitis with hydrocephalus, noted in a fraction of these animals [14]. A study also detected LV in half of the human intrauterine fetal death cases investigated by IHC—the virus being present in the brain and the placenta of investigated cases [13]. LV was diagnosed in nine out of 10 human cases with hydrocephalus and in one out of 18 trisomy 21 controls by IHC [12]. All these observations support the hypothesis that the group of picornaviruses identified as LV and the LV related viruses have neurotropic features in its repertoire.

We have earlier reported that specimens from patients suffering from intrauterine fetal death and malformations had been confirmed positive by PCR [12, 34, 35]. We have subsequently made unsuccessful attempts to confirm these results by sequencing the PCR product using a variety of PCR designed for detection of LV [35, 36]. Brain tissue and cerebrospinal fluid from patients with neurodegenerative diseases, including patients diagnosed with AD, have also repeatedly been found PCR negative (Bo Niklasson, unpublished observations).

The sensitivity and specificity of any diagnostic PCR assay depends on the sequence information available from sequenced viruses representing the genetic variation for the viral disease to be diagnosed. When a new virus is discovered this information by definition is limited, affecting the interpretation of primarily negative results. Presence of a virus can be confirmed by PCR but absence of PCR confirmation does not exclude presence of virus.

A polyclonal antiserum raised against a viral protein such as the rabbit antisera used in the IHC in the present study is expected to react with the virus strain used for immunization and most often also with closely related viruses.

We here thus report the potential findings of LV presence in patients with AD patients exclusively and not in controls, with observations demonstrating the infectious agent in significant amounts at the site of tissue damage in the limbic cortex—the amyloid plaque being the hallmark of AD. In addition, a small number of patients receiving anti-picornavirus treatment showed a positive response on cognitive functions [37].

These intriguing findings, if true, have a fundamental impact on most aspects of AD, but must at this time be interpreted with caution.

## CONFLICT OF INTEREST

The authors have no conflict of interest to report.
